# Polymorphisms in DNA Repair Genes (*APEX1*, *XPD*, *XRCC1* and *XRCC3*) and Risk of Preeclampsia in a Mexican Mestizo Population

**DOI:** 10.3390/ijms15034273

**Published:** 2014-03-11

**Authors:** Ada Sandoval-Carrillo, Edna M. Méndez-Hernández, Fernando Vazquez-Alaniz, Marisela Aguilar-Durán, Alfredo Téllez-Valencia, Marcelo Barraza-Salas, Francisco X. Castellanos-Juárez, Osmel La Llave-León, José M. Salas-Pacheco

**Affiliations:** 1 Institute for Scientific Research, Juarez University of Durango State, 34000 Durango, Mexico; E-Mails: adda-sandoval@hotmail.com (A.S.-C.); aguilarduran_marisela@hotmail.com (M.A.-D.); xavier_castellanos@hotmail.com (F.X.C.-J.); ollave56@yahoo.es (O.L.L.-L.); 2 Faculty of Medicine and Nutrition, Juarez University of Durango State, 34000 Durango, Mexico; E-Mails: edna_madai@hotmail.com (E.M.M.-H.); tellezalfredo@gmail.com (A.T.-V.); sbmaj30@hotmail.com (M.B.-S.); 3 General Hospital of Durango, Secretary of Health, Durango, 34000 Durango, Mexico; E-Mail: feralaniz1@hotmail.com

**Keywords:** preeclampsia, polymorphisms, *APEX1*, *XPD*, *XRCC1*, *XRCC3*

## Abstract

Variations in genes involved in DNA repair systems have been proposed as risk factors for the development of preeclampsia (PE). We conducted a case-control study to investigate the association of Human apurinic/apyrimidinic (AP) endonuclease (*APEX1*) Asp148Glu (rs1130409), Xeroderma Pigmentosum group D (*XPD*) Lys751Gln (rs13181), X-ray repair cross-complementing group 1 (*XRCC*) Arg399Gln (rs25487) and X-ray repair cross-complementing group 3 (*XRCC3*) Thr241Met (rs861539) polymorphisms with PE in a Mexican population. Samples of 202 cases and 350 controls were genotyped using RTPCR. Association analyses based on a χ^2^ test and binary logistic regression were performed to determine the odds ratio (OR) and a 95% confidence interval (95% CI) for each polymorphism. The allelic frequencies of *APEX1* Asp148Glu polymorphism showed statistical significant differences between preeclamptic and normal women (*p* = 0.036). Although neither of the polymorphisms proved to be a risk factor for the disease, the *APEX1* Asp148Glu polymorphism showed a tendency of association (OR: 1.74, 95% CI = 0.96–3.14) and a significant trend (*p* for trend = 0.048). A subgroup analyses revealed differences in the allelic frequencies of *APEX1* Asp148Glu polymorphism between women with mild preeclampsia and severe preeclampsia (*p* = 0.035). In conclusion, our results reveal no association between *XPD* Lys751Gln, *XRCC* Arg399Gln and *XRCC3* Thr241Met polymorphisms and the risk of PE in a Mexican mestizo population; however, the results in the *APEX1* Asp148Glu polymorphism suggest the need for future studies using a larger sample size.

## 1. Introduction

Preeclampsia (PE) is a hypertensive multisystemic disorder unique to humans, affecting approximately 10% of all pregnancies with a slightly higher incidence in developing countries. It is a major cause of maternal death and is responsible for high rates of fetal morbidity and mortality [1]. PE is characterized by *de novo* hypertension (two blood pressure measurements ≥140/90 mm Hg) and proteinuria (>300 mg/24 h) that develops after 20 weeks of gestation in a formerly normotensive woman [2]. The pathophysiology is characterized by abnormal vascular response to placentation that is associated with increased systemic vascular resistance, enhanced platelet aggregation, activation of the coagulation system, and endothelial cell dysfunction [3].

Increasing evidence suggests that oxidative stress plays an important role in the pathogenesis of PE [4,5]. In healthy pregnancies, a balance is maintained between lipid peroxides and anti-oxidative processes. In contrast, PE pregnancies are characterized by an imbalance in these processes leading to an increase in oxidative stress [6–8]. Oxygen concentrations at the fetal-maternal interface fluctuate during pregnancy as a consequence of the vascular remodeling within the tissues of the uterus [9]. Infection/inflammation, intense tissue remodeling, and changes in vascular perfusion generate reactive oxygen species (ROS)—including O_2_^−^, hydroxyl radical, and hydrogen peroxide (H_2_O_2_)—all of which are capable of damaging nucleic acids, proteins, and lipids if levels of these ROS overwhelm the intracellular anti-oxidative defenses [10].

Most damaged DNA can be removed and recovered through pathways carried by DNA repairing enzymes. The normal function of these enzymes is, therefore, important for the maintenance of genomic integrity [11]. A variety of DNA repair processes such as base excision repair (BER), nucleotide excision repair (NER) and mismatch and double-strand break repairs have evolved to perform critical repair functions. DNA repair deficiency has been linked to mutations in some genes that result in a complete loss of DNA repair protein function, whereas more subtle differences in repair capacity occur through the inheritance of polymorphisms within genes [12]. Various DNA repair genes carry genetic polymorphisms with potential to modulate gene function and alter DNA repair capacity [13].

Human apurinic/apyrimidinic (AP) endonuclease (*APEX1*) gene is located at chromosome 14q11.2 and codifies one of the main enzymes in BER pathway, which accounts for nearly all of the abasic site cleavage activity observed in cellular extracts [14]. APEX1 cleaves the phosphodiester backbone immediately at the 50 of abasic site, via hydrolytic mechanism, in order to generate a single strand DNA break leaving a 30-hydroxyl and 50-deoxyribose phosphate terminus [15]. The *APEX1* Asp148Glu (rs1130409) polymorphism have been reported to have higher sensitivity to ionizing radiation [16], and some authors describe the association of the allele G with prostate cancer [17,18].

The excision repair cross-complementing rodent repair deficiency, group 2 (*ERCC2*) gene, also called the xeroderma pigmentosum group D (*XPD*) gene, is located at chromosome 19q13.3. The XPD protein is essential in the NER pathway. This enzyme has a dual role: (i) uncoiling the double helix at the site of DNA lesions and (ii) transcription [19]. Recently, several studies have shown that variant alleles of polymorphism for *XPD* Lys751Gln (rs13181) has been associated with increased DNA adduct levels [20–22] and with reduced capacity to DNA repair [23] and with cancer risk [24–26].

The X-ray repair cross-complementing group 1 (*XRCC*) gene is located at 19q13.2 and produce multidomain protein that acts as a scaffolding intermediate by interacting with Ligase III, DNA polymerase-b and poly-ADPribose polymerase (PARP) [27,28]. XRCC1 protein plays a crucial role in the coordination of two overlapping repair pathways, BER and single strand break repair (SSBR) [29]. Substitutions of Arg194Trp and Arg399Gln (rs25487) may alter the function of the XRCC1 protein [30,31].

The X-ray repair cross-complementing group 3 (*XRCC3*) gene is located at chromosome 19q13.3 and is a member of the RecA/Rad51-related protein complex responsible for the homologous recombinational repair (HRR) of double-strand DNA and is necessary for the stability of the genome [32–34]. The C > T transition is the most often occurring polymorphism in the *XRCC3* gene at codon 241, causing an amino acid change (Thr to Met, rs861539) [35]. Carriers of the Met allele showed a relatively high DNA adduct level in lymphocytes, which could be associated with reduced DNA repair capacity [21,36].

At date, very few studies analyze the possible association between this polymorphisms and risk of preeclampsia [37,38]. In the present study we aimed to investigate the association between *APEX1* Asp148Glu, *XPD* Lys751Gln, *XRCC1* Arg399Gln and *XRCC3* Thr241Met polymorphisms and the risk of preeclampsia in a Mexican population.

## 2. Results and Discussion

### 2.1. Results

A total of 202 cases and 350 controls were included in this study. Of 202 cases, 32 (15.84%), 142 (70.29%) and 28 (13.86%) presented mild PE, severe PE and eclampsia, respectively. Table 1 shows the clinical characteristics. As expected, a difference in pathognomonic variables was observed. Furthermore, we found differences between groups at weeks of pregnancy (*p* < 0.001, Table 1).

Allelic and genotypic frequencies of *APEX1* Asp148Glu, *XPD* Lys751Gln, *XRCC1* Arg399Gln and *XRCC3* Thr241Met polymorphisms are show in Table 2. All polymorphisms were in HWE. The variant allele frequencies of *APEX1* Asp148Glu, *XPD* Lys751Gln and *XRCC3* polymorphisms in our control group were consistent with our previous studies [39]. The allelic frequencies of *APEX1* Asp148Glu showed statistical significant differences between groups (*p* = 0.036). However, these differences were not observed in the genotypic frequencies (*p* = 0.143). No differences in allelic or genotypic frequencies between cases and controls were observed in any of the others polymorphisms tested (Table 2, *p* > 0.05).

A subgroup analyses (mild and severe preeclampsia) revealed no differences in genotypic frequencies of *APEX1* Asp148Glu, *XPD* Lys751Gln, *XRCC1* Arg399Gln and *XRCC3* Thr241Met polymorphisms between women with severe preeclampsia and mild preeclampsia (Table 2). Regarding allelic frequencies, only statistical significant differences between mild PE and severe PE in the *APEX1* Asp148Glu polymorphism were found (*p* = 0.035, Table 2). Additionally, the mild and severe PE groups were tested against their respective control groups, however, statistically significant differences were not found (data no shown).

The risk of preeclampsia by the presence of these polymorphisms was determined. A logistic regression model adjusted for age was used and the results of Table 3 showed that any of the polymorphisms are a risk factor for the disease; however, the *APEX1* Asp148Glu polymorphism showed a tendency of association (OR: 1.74, 95% CI = 0.96–3.14). Furthermore, a significant trend was observed (*p* for trend = 0.048).

### 2.2. Discussion

One of the main features of preeclampsia is an inadequate trophoblast cell invasion that leads to an incomplete remodeling of the spiral artery, reduction in utero-placental perfusion, and a state of placental ischemia. A factor known to be increased in response to placental ischemia is the production of ROS. When ROS production exceeds the capacity of detoxification, they can cause oxidative damage to DNA [29]. The biological importance of oxidative DNA damage remains to be determined, but it is reasonable to suggest that such DNA damage could affect the transcriptional regulation of placental genes involved in implantation and placentation and/or cell fate decisions (apoptosis), thereby contributing to the pathogenesis of PE. Recently, Tadesse *et al.* [40] demonstrated that oxidative stress-induced DNA damage and repair is present in higher amounts in the placentas of women with PE *vs.* gestational age matched normotensive controls.

DNA repair mechanisms are in place to protect against such damage and imply a role for DNA repair genes in the response of the placenta to ischemia. Oxidative DNA damage is repaired by both BER and NER mechanisms [41]. Therefore, genetic polymorphism in BER and NER genes may influence individual variations in DNA repair capacity, which may be associated with risk of developing PE.

The majority of work has focused on evaluate genes involved in antioxidant defense systems rather than DNA repair systems. While some studies suggest the association between polymorphisms of genes involved in antioxidant systems and preeclampsia [42], others have found no association [43]. Specifically, in a Mexican population, data from our laboratory suggest that a *GSTT1* null polymorphism might be associated with preeclampsia and that this risk increases with the combination of both *GSTT1* and *GSTM1* null polymorphisms [44].

On the other hand, to date, very few studies evaluate the relationship between polymorphisms in DNA repair genes and the risk of PE. Vural *et al.* [38] evaluate polymorphisms in *APEX1*, *XRCC1* and *XPD* in a Turkish population. Although they report that none of the studied polymorphisms has been associated with the risk of PE, they suggest that their results need to be taken as preliminaries due to relatively small sample size. Furthermore, Saadat *et al.* [37] investigate the association between the genetic polymorphisms of *XRCC1* and XRCC7 and risk of PE in an Iranian population. They analyze 151 patients with preeclampsia and their results suggest that the 399Gln allele of the *XRCC1* is a significant risk factor for PE development.

In this study, we analyze the relation between *APEX1* Asp148Glu, *XPD* Lys751Gln, *XRCC1* Arg399Gln and *XRCC3* Thr241Met polymorphisms and the risk of PE in a Mexican population. Although our results showed no association between *APEX1* Asp148Glu and PE like previously reported by Vural *et al.* [38], our data showed a tendency of association (OR: 1.74, 95% CI = 0.96–3.14) and a significant trend (*p* for trend = 0.048). These results, together with the statistical significant difference observed in the allelic frequency of this polymorphism between women with mild preeclampsia and severe preeclampsia (*p* = 0.035), suggest a need for future studies with a larger sample to elucidate the role of this polymorphism in the PE in our population.

Our results in the other polymorphisms suggest no association with the risk of the PE. These results are in disagreement with Saadat *et al.* [37], but are in agreement with the reported by Vural *et al.* [38], in the *XRCC1* Arg399Gln polymorphism. With respect to the *XPD* Lys751Gln polymorphism our results are in agreement with Vural *et al.* [38]. Finally, to our understanding any studies has analyzed the association between the *XRCC3* Thr241Met polymorphism and the risk to PE; the results of this study are the first to suggest no association.

The limitations of our study include the lack of oxidative damage measurements in blood samples or placenta that might suggest a possible association between the levels of DNA damage, the genotypes analyzed and PE. Furthermore, our study had limited statistical power because of the low frequency of some of the variants genotypes.

## 3. Experimental Section

### 3.1. Subjects and Study Design

This case-control study was conducted between March 2011 and September 2013. The study was approved by the Ethics Investigation Committee in the Hospital General of the Health Secretary of Durango, Mexico, in accordance with the Code of Ethics of the Declaration of Helsinki. Signed informed consent was obtained from all patients and controls before participation in the study. We recruited 202 women diagnosed with preeclampsia (cases) and 350 normoevolutive pregnant women (controls). The inclusion criteria were all those women diagnosed with mild PE (blood pressure (BP) ≥ 140/90 mm Hg and proteinuria ≥ 30 mg/dL), severe PE (BP ≥ 160/110 mm Hg and proteinuria ≥ 2000 mg/dL) and eclampsia (defined as occurrence, in a woman with preeclampsia, of seizures that cannot be attributed to other causes). Control group was conformed by healthy pregnant women attending at the same hospital; without hypertensive, pathological or metabolic disorders during pregnancy. The control group was followed to corroborate the normality of the blood pressure values.

### 3.2. DNA Extraction and Genotyping of Samples

Genomic DNA from each individual was obtained from blood samples obtained by venous puncture using the QIAamp DNA Blood extraction kit (Qiagen, Hilden, Germany). The concentration and quality were analyzed in a NanoDrop 2000 (Thermo Scientific, Wilmington, DE, USA). The single nucleotide polymorphisms were analyzed using a 48-well plate StepOne Real Time PCR system (Applied Biosystems, Carlsbad, CA, USA). A typical RT-PCR reaction contained 10 ng of genomic DNA, 0.625 μL of 40X Taqman SNP genotyping assay and 5.0 μL of Taqman genotyping Master Mix (Applied Biosystems, Foster City, CA, USA). The predesigned assays were C___8921503_10, C___3145033_10, C____622564_10 and C___8901525_10 (rs1130409, rs13181, rs25487 and rs861539, respectively; Applied Biosystems, Foster City, CA, USA). The amplification conditions were initial denaturation at 95 °C for 10 min, 92 °C during 15 s, and 60 °C for 90 s (42 cycles) with one additional cycle at 60 °C during 30 s. Samples were processed by duplicate.

### 3.3. Statistical Analysis

The clinical characteristics were expressed as mean and were compared by using the Student’s *t*-test. The allele and genotype frequencies were calculated by direct counting. Deviation from the Hardy-Weinberg equilibrium (HWE) constant was tested using a χ^2^ test with 1 degree of freedom. The differences of distributions of the polymorphisms were performed by χ^2^ analysis using SPSS software (version 15.0; SPSS Inc., Chicago, IL, USA); *p* < 0.05 was considered statistically significant. Odds ratio was calculated from allelic and genotype frequencies with 95% confidence intervals (95% CI) using the SNPstats software program (2006, Catalan Institute of Oncology, Barcelona, Spain).

## 4. Conclusions

Although our results suggest that there is no an association between *XPD* Lys751Gln, *XRCC* Arg399Gln and *XRCC3* Thr241Met polymorphisms and the risk of PE, the results in the *APEX1* Asp148Glu polymorphism suggest the needed of future studies using a larger sample size. Furthermore, studies in other regions from Mexico also are needed to confirm our findings. Silva-Zolezzi *et al.* [45] described genetic differences among Mexican Mestizos from different regions of Mexico, mainly due to differences in Amerindian and European contributions; samples from central regions were closer to Amerindian Zapotecos, while samples from northern regions were located closer to Europeans.

## Figures and Tables

**Table 1.** Clinical characteristics of preeclamptic (cases) and healthy pregnant women (controls).

Clinical features	Cases, *n* = 202	Controls, *n* = 350	*p*-value
Age (years) a	24.53 (7.70)	23.57 (6.57)	0.108 b
Weeks of pregnancy a	35.92 (4.75)	37.97 (3.82)	<0.001 b
Number of pregnancies a	2.38 (2.20)	2.24 (1.44)	0.353 b
Systolic blood pressure (mm Hg) a	157.96 (18.71)	109.26 (10.27)	<0.001 b
Diastolic blood pressure (mm Hg) a	102.75 (13.19)	68.82 (8.98)	<0.001 b
Mean arterial pressure (mm Hg) a	121.17 (13.34)	82.18 (8.50)	<0.001 b

a Media (± Standard deviation);

b Independent sample *T* test.

**Table 2.** Allele and genotype frequencies of *APEX1* Asp148Glu, *XPD* Lys751Gln, *XRCC1* Arg399Gln and *XRCC3* Thr241Met polymorphisms in preeclamptic (cases), healthy pregnant women (controls) and mild and severe preeclamptic women subgroups.

Polymorphisms	Cases, *n* = 202	Controls, *n* = 350	*p*-value	Mild PE, *n* (%)	Severe PE, *n* (%)	*p*-value
*APEX1* Asp148Glu *						

Asp	0.61	0.68	0.036 a	44 (70.96)	158 (56.42)	0.035 a,b
Glu	0.39	0.32		18 (29.03)	122 (43.57)	

Asp/Asp	0.40	0.49	0.143 a	16 (51.61)	49 (35)	0.140 a,b
Asp/Glu	0.41	0.38		12 (38.7)	60 (42.85)	
Glu/Glu	0.19	0.13		3 (9.67)	31 (22.14)	

*XPD* Lys751Gln						

Lys	0.79	0.80	0.662 a	51 (82.25)	222 (78.16)	0.475 a,b
Gln	0.21	0.20		11 (17.74)	62 (21.83)	

Lys/Lys	0.61	0.63	0.900 a	22 (70.96)	85 (59.85)	0.284 a,b
Lys/Gln	0.35	0.34		7 (22.58)	52 (36.61)	
Gln/Gln	0.04	0.03		2 (6.45)	5 (3.52)	

*XRCC1* Arg399Gln						

Arg	0.75	0.74	0.514 a	48 (75)	212(75.17)	0.976 a,b
Gln	0.25	0.26		16 (25)	70 (24.82)	

Arg/Arg	0.58	0.56	0.816 a	17 (53.12)	83 (58.86)	0.352 a,b
Arg/Gln	0.34	0.36		14 (43.75)	46 (32.62)	
Gln/Gln	0.07	0.08		1 (3.12)	12 (8.51)	

*XRCC3* Thr241Met						

Thr	0.86	0.83	0.211 a	57 (89.06)	231 (84.92)	0.395 a,b
Met	0.14	0.17		7 (10.93)	41 (15.07)	

Thr/Thr	0.73	0.67	0.276 a	26 (81.25)	97 (71.32)	0.344 a,b
Thr/Met	0.24	0.31		5 (15.62)	37 (27.2)	
Met/Met	0.02	0.02		1 (3.12)	2 (1.47)	

a *Pearson’s Chi*-*squared* is significant at *p* ≤ 0.05;

b The test was performed between mild PE and severe PE groups;

* The control group for this polymorphism was integrated by 204 women.

**Table 3.** Polymorphisms association with preeclampsia.

Polymorphisms	Cases, *n*	Controls, *n*	OR	95% CI	*p*-value
*APEX1* Asp148Glu					

Asp/Asp	80	98	1.00	(referent)	
Asp/Glu	82	77	1.26	0.84–1.97	0.166
Glu/Glu	37	26	1.74	0.96–3.14	
*p value for trend*					0.048

*XPD* Lys751Gln					

Lys/Lys	123	220	1.00	(referent)	
Lys/Gln	70	118	1.11	0.72–1.707	0.868
Gln/Gln	8	12	1.15	0.413–3.227	
*p value for trend*					0.655

*XRCC1* Arg399Gln					

Arg/Arg	118	195	1.00	(referent)	
Arg/Gln	69	126	0.939	0.608–1.45	0.854
Gln/Gln	15	29	0.818	0.393–1.70	
*p value for trend*					0.528

*XRCC3* Thr241Met					

Thr/Thr	144	235	1.00	(referent)	
Thr/Met	48	108	0.76	0.487–1.195	0.211
Met/Met	4	6	4.047	0.443–36.972	
*p value for trend*					0.197

Abbreviations: CI, confidence interval; OR, odds ratio.
